# The Impact of Financial Redundancy on Corporate Social Responsibility Performance: Evidence From Chinese Listed Firms

**DOI:** 10.3389/fpsyg.2022.882731

**Published:** 2022-05-19

**Authors:** Ling He, Shengdao Gan, Tingyong Zhong

**Affiliations:** ^1^School of Business, Sichuan University, Chengdu, China; ^2^School of Accounting, Chongqing Technology and Business University, Chongqing, China

**Keywords:** financial redundancy, corporate social responsibility, motivation, consequences, managerial career concerns, market competition

## Abstract

This study examines the impact of financial redundancy on corporate social responsibility (CSR) based on a sample of Chinese listed firms from 2010 to 2020. The results indicate that financial redundancy has a significant positive effect on CSR. However, financially redundant resources are not balanced in terms of how they encourage firms to undertake different dimensions of social responsibility; specifically, firms actively take social responsibility toward shareholders and the public but take less responsibility for employees and the environment. The incentive for firms with financially redundant resources to promote CSR initiatives is attributable to their high level of social awareness and pursuit of reputation. Consistent with their motives, our economic consequence analysis reveals that the incremental effect of CSR driven by financial redundancy improves corporate reputation but has no enhancement effect on corporate performance. Finally, our extended analysis reveals that the relative impact of financial redundancy on CSR depends on several organizational variables that influence a firm’s preferences for CSR investments. The positive impact of financial redundancy on CSR is stronger among firms with high managerial career concerns and firms in regions with high market competition. This research provides a necessary structure for future CSR studies to follow. By delving deeply into the relationship between financial redundancy and CSR, it enables scholars to better address the critical management question of whether wealthy firms do more good for society compared to those that are less wealthy.

## Introduction

With the frequent occurrence of natural disasters, environmental pollution, issues with employee rights protection, problems with food safety, instances of tax evasion, and other incidents in China, the call for enterprises to assume social responsibility has increased dramatically. China has launched a number of initiatives to promote corporate social responsibility (CSR) development. However, in contrast to the increasing number of laws and regulations, firms are taking socially responsible actions in a high-profile manner while at the same time committing socially irresponsible acts. For example, in 2010, Foxconn donated resources to earthquake-stricken areas; however, in the same year, 13 Foxconn employees jumped from a building. Similarly, Vanke, which won the “Best Corporate Citizen award in China,” has repeatedly been found to have product quality problems. The lack of CSR in various industries is becoming a common phenomenon, which seems to indicate that the manifestation of CSR in China is complex and heterogeneous. As a socialist country, China’s CSR performance is among the lowest of all global economies. Therefore, it is necessary to explore firms’ motivations for undertaking CSR in the context of China. Such research will have important theoretical and practical significance for expanding research on CSR and promoting China’s sustainable growth.

CSR pertains to actions that a firm undertakes beyond the firm’s interests and legal requirements, and can refer to employees, the environment, suppliers, consumers, the public, and other parties (Oh et al., 2011; Marakova et al., 2021). Research on the driving factors of CSR has always been a key topic in academia. Early studies have observed several antecedents of CSR, including external factors, such as public attention (Zyglidopoulos et al., 2012; Cheng and Liu, 2018; El Ghoul et al., 2019) and institutional environment (Flammer, 2015; Han et al., 2022); and internal factors, such as corporate governance (Hong et al., 2016), ownership structure (Oh et al., 2011; Li et al., 2021), executives characteristics (Zu and Song, 2009; Fabrizi et al., 2014), and board characteristics (Chang et al., 2017; Ardito et al., 2021). It must be recognized that firms face financial costs for their socially responsible investments, and adequate funding is a basic prerequisite for enterprises to undertake social responsibility (Leong and Yang, 2021; Xiao et al., 2021).

Socially responsible investment is one of the investment behaviors of firms. Firms have a hierarchy of investment priorities, and prior research has suggested that firms place their core business investment needs at the top of this hierarchy and CSR lower down (Waddock and Graves, 1997; Sun et al., 2021). One critical implication of this is that firms’ CSR-related actions may depend on whether their core business investment needs have already been met; that is, firms will undertake social responsibility only after satisfying normal corporate operations and thereafter having surplus resources.

With the development of China’s capital market, a particular financial phenomenon has appeared in the financial practice of firms: that is, firms often hoard large amounts of cash while reducing interest-bearing debt. Studies have defined this as financial redundancy, which depicts a financial status in which the firm retains liquid capital that exceeds its operating demands and has a low interest-bearing debt ratio (Myers and Majluf, 1984; Alessandri et al., 2014). Financial redundancy—a major form of organizational redundancy—is a financial resource that the organization has acquired but not designated for necessary consumption, and that can be freely used by managers. According to our survey, more than half of listed firms in China have hoarded large amounts of financially redundant resources, and this has become a normalized financial phenomenon among Chinese firms. Financially redundant resources are those that exist beyond resources needed for enterprises to maintain operations; they can be used to meet the other requirements of stakeholders, and their attributes to be allocated and the consumption characteristics of resources to undertake social responsibility are complementary in function. However, few studies have focused on this point. Thus, a question remains as to whether firms that are rich in financial resources engage in more social responsibility compared to those that are not.

Theoretically, the impact of financial redundancy on CSR is ambiguous. On the one hand, financial redundancy can solve firms’ financial problems; in addition, it encourages strategic behavior, eases adaptation to new environments, fosters long-term thinking, and enables the exploration of uncertain investment opportunities. Sufficient financially redundant resources play the role of a “resource buffer pool” when firms face resource bottlenecks and ensure the sustainability and stability of the CSR investment. On the other hand, the high flexibility of financially redundant resource makes firms extremely vulnerable to agency problems (Shahzad et al., 2016; Suzuki, 2018). Excessive redundant resources will weaken firms’ internal control, leading managers to allocate redundant resources to low-risk investment activities that are beneficial to them personally, thereby avoiding CSR investment projects that increase corporate risks, have a long profit cycle, and are slow to yield results. Consequently, what is the effect of financial redundancy on CSR? What is the mechanical path therein? Does the effect differ under diverse conditions? To explore these issues, we select data from Chinese listed firms from 2010 to 2020 to explore the relationship between financial redundancy and CSR in this paper, as well as the heterogeneity of the relationship in different environments, and to further investigate the motivations to undertake CSR and the economic consequences of doing so.

Our work contributes to the literature in the following aspects. First, this study expands on previous research regarding the strategic motivation of CSR. The results show that firms will use tactical behaviors to take on the part of social responsibilities that are conducive to the firm’s short-term development, while avoiding social responsibility projects that require long-term investment and are slow to yield results. This finding provides a new perspective on the strategic motivation for CSR. Second, our study enriches the relevant literature on CSR. The early literature mainly studied the influencing factors of CSR from a single dimension and lacked a classification system to explore the motivations for undertaking social responsibility in different dimensions (Flammer, 2015). Social responsibility has now become a multidimensional concept. Hence, this paper focuses on five aspects to study the impact of financial redundancy on CSR in different dimensions, which can deepen understanding of the “black box” of CSR. Third, this paper expands the literature on financial status and CSR. Existing studies have discussed the factors influencing CSR performance from a financial perspective (Waddock and Graves, 1997; Lin et al., 2019; Hou et al., 2021), overlooking the transmission mechanisms in depth. Our study fills this gap by examining the motivations and economic consequences of firms with financially redundant resources engaging in CSR initiatives, and clarifying that the involvement of such firms in CSR initiatives is driven by a combination of social awareness and reputation-seeking motives. This finding augments the relevant literature empirically and theoretically, especially in the context of emerging countries. Finally, our results show that the relative impact of financial redundancy on CSR depends on several variables that influence the structure of a firm’s investment priorities (managerial career concerns and market competition). These results extend past findings that have documented a link between these factors and CSR itself, suggesting that they not only influence a firm’s absolute level of CSR but also affect its willingness to change CSR strategy under the influence of internal financial resources.

## Literature Review

### Reasons for Financial Redundancy

There are several reasons why for the existence of the phenomenon of financial redundancy in Chinese listed firms. (1) Corporate life cycle. When the firm is in the early stages of entrepreneurship, it may have debt financing constraints and hold large amounts of cash in anticipation of investment opportunities. Firms in a more mature stage have entered a state of relatively stable and continuous growth, and tend to hoard large amounts of monetary funds. (2) Traditional culture. China’s traditional culture emphasizes risk avoidance and preparedness, and these traits when transferred to corporate financial decisions are reflected as financial soundness and risk aversion. The traditional culture makes corporate decision-making toward avoiding debt repayment risks, using less financial leverage, and relying on internal financing. Under the long-term influence of this business strategy, firms will be prone to the phenomenon of financial redundancy. (3) Management’s pursuit of personal interests. To pursue personal interests, managers may reduce or avoid paying dividends, which causes large amounts of funds within the firm, and the long-term accumulation of funds eventually leads to financial redundancy. (4) Some listed firms achieve equity refinancing conditions through earnings management and fabricated investment projects and then carry out financial activities such as supplementing working capital and repaying debt after obtaining funds, which also causes the phenomenon of financial redundancy.

### The Linkage of Financial Redundancy With Other Financial Concepts

The concept of financial redundancy as used in this paper is both related to and distinct from those of capital structure, working capital, and free cash flow. The capital structure mainly reflects the proportional allocation on the right-hand side of the balance sheet; that is, the relationship between long-term liabilities and owners’ equity. Financial redundancy proposed in this paper refers to the financial phenomenon in which the firm’s liquidity funds are greater than its liabilities. Financial redundancy can be concentrated on both the left and right sides of the balance sheet, and specifically considers the difference between assets and liabilities, which can more intuitively measure the financial risk of the firm than capital structure. Working capital is the difference between current assets and current liabilities, which reflects the short-term financial status of the firm. However, financial redundancy does not directly compare the size of the firm’s current assets and current liabilities; it more accurately reflects, to a certain extent, the medium- and long-term financial position of the firm from the perspective of the level of cash holdings and the debt commitment level. The term “free cash flow” was first coined by Jensen in 1986. After observing highly profitable oil firms for about 20 years, he found that firms did not pay cash dividends to shareholders and squandered the remaining cash on inefficient investment activities. He thus termed the cash that should be returned to shareholders but remains held in the firm free cash flow. Financial redundancy represents a financial situation in which a firm has good financial performance, relatively high free cash flow, and lower dividends. It can also be referred to as free cash flow increment, which is a kind of financial redundancy formed *via* the accumulation of free cash flow within the firm over many years. However, free cash flow only focuses on cash flow, while financial redundancy takes into account both cash flow and stock, and integrates the firm’s cash holdings and liabilities. Therefore, it is better than free cash flow as an indicator to describe a firm’s medium- and long-term financial status.

### Motivations for Corporate Social Responsibility

Corporate social responsibility refers to the responsibility of enterprises to take into account the government, suppliers, customers, employees, and other stakeholders, as well as duties related to environmental protection. Existing literature has conducted rich explorations on the motivations of firms to undertake social responsibility. These motivations can be divided into altruistic, self-interest, and strategic.

Altruistic motivation refers to firms engaging in CSR behavior based on moral and ethical. When it comes to altruistic motives, CSR is seen as a true attempt by firms to solve social problems and improve overall social welfare through socially responsible behaviors. Previous studies have verified the altruistic motivation of social responsibility based on legitimacy theory and social contract theory (Ferrell et al., 2016; Rossi et al., 2021).

Self-interest motivation holds that firms take on social responsibility as a tool to seek personal benefits and as a means to cover up or whitewash their improper behaviors. Drawing on opportunistic tool theory, shareholder primacy theory, attribution theory, and principal-agent theory, scholars have conducted empirical studies on this motivation from the perspectives of earnings management, tax evasion, negative events, and political connection. For example, Prior et al. (2008) found that enterprises undertake social responsibility in order to conceal earnings management behavior, while Kotchen and Moon (2012) demonstrated that firms may simply use CSR to whitewash corporate irresponsibility.

Strategic motivation can be mainly explained based on resource dependence theory, stakeholder theory, competitive advantage theory, and strategic choice theory. It is believed that firms can improve their strategic position and obtain various strategic resources by assuming social responsibility. Dhaliwal et al. (2014) and Marakova et al. (2021) contended that CSR activities can not only build a positive corporate image and enhance competitive advantage but also provide key resources for the sustainable development of firms by meeting the requirements of various stakeholders.

### Financial Resources and Corporate Social Responsibility

As stakeholders pay increasing attention to CSR, some scholars have begun to explore the driving factors of CSR from the perspective of financial resources. However, evidence on the impact of corporate financial resources on CSR is mixed. Based on the resource dependence theory, most scholars have agreed that higher financial performance leads enterprises to take on more social responsibilities. For example, Lin et al. (2019) asserted that better financial performance of firms leads to higher CSR engagement. Waddock and Graves (1997) claimed that firms with high profitability can invest more in CSR compared to firms with lower profits. Nevertheless, some scholars have stated that financial resources do not make a positive contribution to CSR activities. For instance, Julian and Ofori-Dankwa (2013) studied the influence of financial resource availability on CSR and found that there are large differences between economies. Taking Ghana as the study subject, they found that firms with more financial resources spend less on CSR. Similarly, Shahzad et al. (2016) highlighted that excessive slack increases managers’ complacency and idleness, resulting in a lack of motivation to undertake CSR. Using data from Nigerian, Boso et al. (2017) showed that increases in financial resource slack are associated with decreases in sustainability spending by firms.

In short, while CSR has been extensively explored and studied, deficiencies remain. First, there is still controversy surrounding the motivations of enterprises to fulfill social responsibilities. CSR is a multidimensional variable, environmental responsibility and charitable donations only reflect one aspect of CSR. Therefore, it is necessary to analyze the various dimensions of social responsibility indicators, as well as the economic consequences of CSR, which can be used to comprehensively evaluate CSR motives from a more detailed perspective. Second, in terms of financial indicators, prior studies have used income statement data to reflect the short-term financial conditions of firms, but these indicators may be undermined by earnings management (Li et al., 2014). Therefore, when studying the relationship between corporate financial resources and CSR, it may be difficult to draw reliable conclusions without excluding the influence of such noise. Third, existing studies have not reached a consensus on the impact of financial resources on CSR. A possible reason for this is that enterprises have different characteristics in allocating financial resources in different capital markets. As Julian and Ofori-Dankwa (2013) and Li et al. (2021) pointed out, institutional differences between developed and developing economies may result in different CSR implications. In addition, existing literature has not analyzed the mechanisms underlying the impact of financial resources on CSR, which may be another reason for the controversial conclusions. Financial redundancy refers to financial resources that exceed the operational requirements of the firm and can be used to meet the other needs of stakeholders. The attributes of financial redundancy need to be allocated are complementary to the resource consumption characteristics of undertaking social responsibility. Financial redundancy has developed into a common financial phenomenon among Chinese listed firms, but few studies have explored the relationship between financial redundancy and CSR in the unique context of China’s socialist market economy, and, in particular, no specific explanation has been given for the mechanistic paths involved.

## Hypothesis Development

Financial redundancy is a type of redundant resource, which is caused by good financial performance, low dividends, and the accumulation of free cash flow within the firm. Financial redundancy plays a decisive role in a firm’s long-term investment decisions. When the degree of financial redundancy is high, it can not only improve the firm’s financial ability but also increase its motivation to undertake social responsibility activities, and ultimately improve CSR performance.

From the perspective of ability, financial redundancy affects the financial strength of enterprises to engage in social responsibility activities; specifically, greater financial redundancy is expected to lead to greater financial support for CSR activities. Enterprises need to pay financial costs to undertake social responsibility initiatives, including direct economic costs and opportunity costs (Sprinkle and Maines, 2010). However, the benefits of CSR come in both monetary and non-monetary forms, and there is a time lag in converting non-monetary benefits into monetary benefits. Since both inputs and outputs of social responsibility need financial support, enterprises can only develop socially responsible activities when they have sufficient financial resources. Financial redundancy implies that the firm has large cash reserves, low debt levels, and low financing costs (Alessandri et al., 2014; Xiao et al., 2021). The higher the degree of financial redundancy, the greater the firm’s remaining discretionary capital. Financially redundant resources can not only provide financial support for CSR but also compensate for the revenue lag when social responsibility is transformed into economic performance. Conversely, when the firm is in financial distress, its economic needs for survival are far higher than its social needs. In such cases firms tend to adopt conservative business strategies and allocate energy and capital to projects that can quickly improve performance (March and Shapira, 1987; Leong and Yang, 2021). This results in enterprises paying less attention to long-term investment-oriented social responsibility activities and decreasing investments in CSR projects.

From the perspective of motivation, financial redundancy can affect the importance enterprises attach to social responsibility. Based on firm-level motivation, firms with financially redundant resources usually have strong financial strength, large scale, and dominant position in the industry, and their behavior is widely scrutinized by outsiders. However, firms that receive more external attention are more vulnerable to stakeholder pressures. As stakeholders place increasing value on CSR, firms may respond to this preference by devoting available resources to CSR (Zyglidopoulos et al., 2012; El Ghoul et al., 2019). In addition, external attention will enhance the supervision effect of the external market. Greater external attention will result in severe penalties for firms that evade their social responsibility or fail to meet public expectations (Sun et al., 2021). Such firms, especially those that have financial redundancy, are likely to suffer reputational damage for not participating in CSR initiatives. Finally, external attention can accelerate the process of transforming CSR into financial performance, encouraging enterprises to fulfill more social responsibility. Thus, firms with high levels of financial redundancy will attract more external attention, which will enhance their motivation to undertake social responsibility driven by the two aspects of incentive and supervision.

In terms of executive motivation, when firms possess high levels of financial redundancy there is ample room for executives to allocate resources to projects that benefit private interests. Malmendier et al. (2011) asserted that executives become overconfident when a firm’s redundant resources exceed a certain level. The higher the level of financial redundancy, the higher the possibility of executive overconfidence, and the higher the executives’ desire to increase reputation and achieve self-achievement. Executives of firms with financially redundant resources pay more attention to spiritual satisfaction after meeting profit demands, and fulfilling social responsibility can satisfy their needs to improve social status, build a good image, and demonstrate their ability (Barnea and Rubin, 2010; Masulis and Reza, 2015). Especially in recent years, China has launched a series of social responsibility awards for outstanding entrepreneurs and most respected firms, which have pushed enterprises to practice greater social responsibility. Therefore, due to the desire for reputation, executives of firms with financially redundant resources tend to use corporate funds to support their social responsibility preferences, so as to achieve a sense of self-achievement and superiority. On the other hand, financial redundancy may result from managers hoarding large amounts of funds within the firm by paying few or no dividends. If managers spend large amounts of funds on on-the-job spending or discretionary expenses will trigger dissatisfaction from investors and shareholders. Therefore, managers have an incentive to find a rational outlet for these redundant resources. Social responsibility can bring reputation to them and also easily receive support from stakeholders, so CSR provides a suitable outlet for these redundant funds.

Therefore, our first hypothesis is drawn from the discussion above, and is stated formally below:

Hypothesis 1: Financial redundancy has a positive effect on CSR performance.

Social responsibility is a multidimensional concept, and firms need to meet the requirements of various stakeholders, including the government, shareholders, consumers, employees, and the public. Excess financial resources will lead to an increase in agency costs and managers’ self-interested motives (John et al., 2017). Different dimensions of social responsibility have different costs and benefits, and the principal-agent problem also widely exists in CSR behavior (Masulis and Reza, 2015). Due to the agency problem, financially redundant firms may face strategic choices when performing various dimensions of social responsibility.

On the one hand, limited resources drive firms to be selective regarding their socially responsible investments. CSR requires management to construct multiple objectives that meet the needs of various stakeholders, but different interest groups have different needs and expectations, and corporate resources are limited. Trade-off theory states that under the condition of limited resources enterprises cannot fully satisfy the demands of every stakeholder; thus, firms will inevitably adjust and weigh the needs of these disparate groups (Krishnamurti et al., 2021). In the face of the needs and pressures of powerful stakeholders, enterprises usually give priority to their social responsibilities, while the social responsibility of other vulnerable stakeholders is deferred or ignored. On the other hand, the costs involved in social responsibility initiatives may result in short-sighted investment behaviors of enterprises. CSR activities are complex processes with high investment, multiple levels, and long cycles; these factors require enterprises to continuously invest large amounts of capital and resources, but without seeing benefits until the next period or a certain period in the future (Waddock and Graves, 1997). Thus, redundant financial resources in firms will create agency problems, which may prompt firms to make short-sighted decisions when making socially responsible investments. Enterprises prefer short-cycle, high-profile, and low-cost CSR practices, and dislike long-term social responsibility because it will reduce corporate value in the short term.

Firms with financially redundant resources actively assume shareholder responsibility not only drive original shareholders to continue to invest in the firm but also attract new shareholders to join the firm, which is conducive to improving the firm’s financing capacity. Firms undertake public responsibility, such as charitable donations and disaster relief. Under the influence of media publicity, these public responsibilities will have a rapid positive impact on firms, which makes corporate executives more inclined to use the firm’s available funds to invest in public responsibility to obtain reputation benefits quickly. Supply chain responsibility includes responsibility to consumers and suppliers. Customers are the firm’s main source of revenue, and suppliers are the firm’s guarantee to enter the market. Firms undertaking supply chain responsibility can directly bring economic inflow and promote the growth of economic profit.

Conversely, compared with other responsibilities, employee responsibility and environmental responsibility are more easily affected by policy regulations, and enterprises lack the initiative to undertake these responsibilities. The power of enterprise employees is relatively weak, and managers may reduce CSR costs by reducing employee compensation to achieve other responsibility goals. Meanwhile, the agency problem caused by financial redundancy will make it difficult to guarantee the rights and interests of employees. Environmental responsibility entails the characteristics of positive externality, long cycle, slow effect, and high cost. For listed firms with abundant financial resources, engaging in environmental investment will crowd out investment funds for other economic projects and reduce short-term performance even if they have significant funds. Motivated by the pursuit of personal benefits, Managers prefer to spend money on social responsibility that can quickly generate revenue inflows and are unwilling to invest in environmental protection. For example, Campbell (2007) found that firms donate large amounts of money to charities but spend little on employees and the environment. Briscese et al. (2021) established a game model to demonstrate that firms compensate for donations by reducing employees’ wages; that is, firms increase their charitable donations by significantly reducing employees’ incomes through an exchange mechanism.

In sum, we believe that the firm is a complex organization that integrates egoism and altruism. On the one hand, it uses egoism to pursue profits; on the other hand, as a social entity, it is afraid of being disconnected from society. Therefore, the firm may show unbalanced in undertaking social responsibility. Combining these arguments, our second hypothesis is formally stated below:

Hypothesis 2: Financial redundancy has an unbalanced effect in promoting enterprises to take on different dimensions of social responsibility, such that enterprises will selectively undertake social responsibility.

## Research Design

### Data

In this study, we collected data on Chinese A-share listed firms from 2010 to 2020. CSR information at the firm level was extracted from Hexun.net^1^. We choose to start our sample from 2010 because Hexun has published CSR data since this date. The website evaluates the social responsibility reports, sustainability reports, and financial reports of all listed firms, and the scores of CSR are given different weights according to the nature of the industry, which can comprehensively and fairly reflect the level of CSR. All financial data were extracted from the China Stock Market and Accounting Research (CSMAR) database and the Chinese Research Data Services (CNRDS) platform. First, we excluded the sample with unavailable or missing data. Second, we eliminated financial, insurance, and securities listed firms, and listed firms subject to special treatment and particular transfer to reduce measurement error. Third, to reduce the potential impact of outliers, all continuous variables were winsorized at the 1% and 99% quantiles. Following these procedures, the final sample comprised 25,044 firm-year observations from 3,310 Chinese listed firms.

### Independent Variable

The independent variable in this paper is financial redundancy (*FEX*). There is no uniform measurement standard for financial redundancy in academia. For example, Julian and Ofori-Dankwa (2013) and Boso et al. (2017) used return on sales, return on equity, and net profit as proxy variables for financial slack resources. Shahzad et al. (2016) calculated excess working capital as a measure of financial slack by subtracting the firm’s current liabilities from its current assets. Leyva-de la Hiz et al. (2019) adopted the current ratio and return on assets as financial slack proxy variables. Previous literature has mainly used simple and general financial indicators to measure financial redundancy. For example, the current ratio only reflects the liquidity capacity of enterprises. Different enterprises have different requirements for asset liquidity due to variations in the nature of their business and their operating conditions. The net profit index only shows the current profitability of the firm, and cannot reflect its financial redundancy. Working capital only reflects a firm’s short-term financial redundancy, not its long-term financial redundancy. Other indicators face similar limitations. Therefore, it is necessary to improve the existing metrics. Referring to the definition of financial redundancy proposed by Myers and Majluf (1984) and Alessandri et al. (2014), financial redundancy refers to the financial status in which the capital is held by the firm exceeds its operating demand and has a low interest-bearing liability ratio. We measure the degree of financial redundancy through the difference between the sum of monetary funds plus trading financial assets and interest-bearing liabilities after the standardization of total assets. Monetary funds comprise the cash holdings accumulated over a long period, and transaction financial assets are financial assets with high liquidity. The sum of the two reflects the number of funds have accumulated by the firm over time. Interest-bearing liabilities indicate the firm’s debt situation. The former minus the latter represents the degree of redundancy in the firm’s discretionary funds available after debt repayment. To eliminate differences in the individual characteristics of firms, total assets are used for standardization. A higher value of this indicator indicates a higher level of financial redundancy in the firm. In addition, firms with a value greater than 0 are considered financially redundant firms, while those with values less than 0 are called non-financially redundant firms.

### Dependent Variable

The dependent variable is CSR. CSR data were mainly obtained from the scores of independent rating agencies. Compared with questionnaire surveys, scoring data can reduce small-sample problems and improve research objectivity and repeatability. The most popular CSR databases are KLD, RNS, and Hexun. However, the KLD database is for United States firms and lacks data for Chinese firms; RKS only uses social responsibility reports to evaluate CSR, but not all firms disclose their social responsibility reports. There is thus a lack of CSR data for firms that do not disclose social responsibility reports. Based on public information, financial reports, social responsibility reports, and sustainability reports, Hexun evaluates the CSR of all listed firms in China. The Hexun score reflects the extent to which firms undertake social responsibility, with higher scores indicating better CSR performance. The total score of Hexun is 100 points. The Hexun CSR score is being increasingly used and recognized as the most authoritative indicator of CSR performance of Chinese listed firms (Hou et al., 2021; Sun et al., 2021). Hence, to analyze the CSR performance of listed firms and avoid sample selection bias, the CSR data were obtained from Hexun. In addition, this paper divides social responsibility into five indicators: shareholder responsibility (*SH*), employee responsibility (*EM*), supply chain responsibility (*SCC*), environmental responsibility (*ENV*), and public responsibility (*SOC*).

### Control Variables

We control for several well-known determinants of CSR based on previous research (Flammer, 2015; Chang et al., 2017; Han et al., 2022). (1) Property rights (*State*). A dummy variable that equals 1 if the controlling shareholder of the firm is state-owned, and 0 otherwise. (2) Firm size (*Size*). The logarithm of total revenue. (3) Ownership concentration (*Top*). The shareholding percentage of the largest shareholder. (4) Firm performance (*Roa*). The return on assets measures the firm’s profitability. (5) Financial leverage (*Lev*). The ratio of liabilities to assets. (6) The growth rate of operating income (*Growth*). The ratio of current year’s revenue increase to last year’s total revenue. (7) Executive compensation (*Salary*). The natural logarithm of the total compensation of the top three executives. (8) CEO-Chairman duality (*Dual*). A dummy variable that equals 1 if a firm’s chairman and CEO are the same people and 0 otherwise. (9) Board size (*Board*). The total number of directors on board. (10) Firm age (*Age*). The logarithm of 1 plus the difference value between the year of the current year and the year when the firm established. Furthermore, we control industry (*Industry*) and year (*Year*) effects.

### Model Building

To investigate whether financial redundancy can promote CSR performance, we conduct the ordinary least square (OLS) regressions model. Considering that CSR has a certain time lag, and at the same time, to further reduce the potential endogenous problems that may exist between the two, social responsibility data lagging one period is applied. The regression model is as follows:


(1) $$C\,S\,R_{i,t+1}=\alpha_{1}+\alpha_{2}\,F\,E\,X_{i,t}+\sum_{j}\alpha_{j}\,C\,o\,n\,t\,r\,o\,l_{i,t}+\varepsilon_{i,t}$$

Where *i* and *t* denote the firm and the year, respectively. *CSR* is the dependent variable, which represents CSR performance. The key independent variable is *FEX*, which is our proxy for financial redundancy, we measure the degree of financial redundancy through the difference between the sum of monetary funds plus trading financial assets and interest-bearing liabilities after the standardization of total assets; *Control* is a vector of control variables, including property rights (*State*), firm size (*Size*), firm age (*Age*), leverage ratio (*Lev*), return on assets (*Roa*), the growth rate of operating income (*Growth*), executive compensation (*Salary*), ownership concentration (*Top*), the total number of directors on board (*Board*), CEO-Chairman duality (*Dual*), as well as industry (*Industry*) and year (*Year*). ε is the error term.

To verify hypothesis 2, financial redundancy has an unbalanced effect on firms undertaking social responsibility projects of different dimensions, we construct the following model:


(2) $$S\,H_{i,t+1}/E\,M_{i,t+1}/S\,C\,C_{i,t+1}/E\,N\,V_{i,t+1}/S\,O\,C_{i,t+1}=\beta_{1}+\beta_{2}\,F\,E\,X_{i,t}+\sum_{j}\beta_{j}\,C\,o\,n\,t\,r\,o\,l_{i,t}+\varepsilon_{i,t}$$

## Results

### Descriptive Statistics

Table 1 summarizes the descriptive statistics. There are big differences in social responsibility scores between different firms, the mean and median of CSR are 23.758 and 21.900, respectively, the median is lower than the mean, suggesting that the level of CSR in China at a relatively low level (Sun et al., 2021). The minimum and maximum values of the five sub-indicators of CSR are far from each other, indicating that the development of social responsibility in China is extremely unbalanced. Correspondingly, the differences in the degree of financial redundancy of various firms are also obvious, with the minimum value of −0.533, and the maximum value of 0.687.

**TABLE 1:** Descriptive statistics of variables.

Variable	N	Mean	Median	Min	Max	SD
CSR	25,044	23.758	21.900	−18.450	90.870	15.666
SH	25,044	13.844	14.560	−12.670	28.190	6.524
SCC	25,044	2.533	1.560	−0.160	15	2.926
EM	25,044	1.376	0	0	20	4.301
SOC	25,044	4.582	4.190	−15	30	4.487
ENV	25,044	1.424	0	0	30	4.632
FEX	25,044	0.030	0.019	−0.533	0.687	0.253
State	25,044	0.367	0	0	1	0.482
Size	25,044	21.415	21.271	18.281	25.485	1.445
Roa	25,044	0.041	0.039	−0.201	0.193	0.054
Lev	25,044	0.419	0.410	0.048	0.876	0.209
Growth	25,044	0.196	0.119	−0.520	2.966	0.443
Top	25,044	0.347	0.330	0.034	0.746	0.152
Salary	25,044	14.321	14.300	12.651	16.268	0.697
Dual	25,044	0.273	0	0	1	0.445
Board	25,044	3.185	3	0	8	0.589
Age	25,044	2.780	2.833	1.386	3.434	0.385

We conduct univariate tests to compare the mean value of CSR between financially redundant firms and non-financially redundant firms. The univariate test results are shown in Table 2. The results show that the mean CSR of the former is significantly higher than the mean CSR of the latter, indicating that firms with more financially redundant resources engage in more CSR activities than firms with less financially redundant resources.

**TABLE 2:** Univariate tests.

	Financially redundant firms		Non-financially redundant firms			
			
Variables	Obs	Mean	Obs	Mean	Mean-Diff	*T*-Test
CSR	13,287	24.577	11,757	22.833	−1.744***	−8.805
State	13,287	0.293	11,757	0.451	0.157***	26.113
Size	13,287	21.868	11,757	21.013	0.855***	48.921
Roa	13,287	0.057	11,757	0.023	−0.035***	−53.215
Lev	13,287	0.300	11,757	0.554	0.253***	120.321
Growth	13,287	0.191	11,757	0.202	0.011*	1.932
Top	13,287	0.346	11,757	0.347	0.001	0.510
Salary	13,287	14.306	11,757	14.337	0.031***	3.494
Dual	13,287	0.319	11,757	0.219	−0.100***	−17.857
Board	13,287	3.118	11,757	3.262	0.144***	19.461
Age	13,287	2.724	11,757	2.843	0.119***	24.700

* and *** stand for the significance levels of 10 and 1%, respectively.

### Baseline Results

The univariate analysis provides us with preliminary evidence on the positive relationship between financial redundancy and CSR. In this section, we perform multivariate regressions to investigate the basic linear results of the impact of financial redundancy on the firm’s overall CSR performance. Column (1) of Table 3 shows the OLS estimation results. The coefficient of *FEX* is positive and significant, which provides strong support for Hypothesis 1. In other words, firms with financially redundant resources invest more resources in social responsibility activities, and their CSR score is higher, which is consistent with our expectation that financial redundancy promote firms to undertake social responsibility. From the perspective of economic significance, it indicates that a one standard deviation increase in the degree of corporate financial redundancy increases the CSR score by 0.028 (=2.654 × 0.253/23.758).

**TABLE 3:** The impact of financial redundancy on CSR.

	(1)	(2)	(3)	(4)	(5)	(6)
Variables	CSR	SH	SCC	EM	ENV	SOC
FEX	2.654***	3.776***	0.071	−0.959***	−1.353***	1.119***
	(5.099)	(19.141)	(0.709)	(−6.209)	(−8.182)	(7.052)
State	1.037***	−0.527***	0.609***	0.371***	0.596***	−0.012
	(4.674)	(−6.520)	(13.855)	(5.641)	(8.573)	(−0.179)
Size	2.205***	0.681***	0.272***	0.447***	0.520***	0.285***
	(24.427)	(19.063)	(15.365)	(16.984)	(17.816)	(10.265)
Roa	64.807***	50.990***	1.501***	3.108***	1.696***	7.510***
	(33.261)	(53.974)	(4.552)	(6.194)	(3.185)	(12.057)
Lev	−5.855***	−3.209***	−0.030	−1.223***	−1.352***	−0.040
	(−8.406)	(−10.627)	(−0.234)	(−6.420)	(−6.409)	(−0.176)
Growth	0.128	0.126	0.114***	−0.087	−0.097*	0.072
	(0.641)	(1.497)	(2.952)	(−1.566)	(−1.728)	(1.042)
Top	4.068***	3.475***	−0.141	−0.093	−0.021	0.847***
	(6.686)	(15.331)	(−1.191)	(−0.505)	(−0.108)	(4.637)
Salary	3.210***	1.151***	0.741***	0.545***	0.457***	0.315***
	(20.634)	(18.939)	(24.933)	(12.052)	(9.552)	(6.653)
Dual	−0.248	−0.012	−0.094***	−0.050	−0.066	−0.025
	(−1.342)	(−0.160)	(−2.693)	(−0.949)	(−1.195)	(−0.419)
Board	0.808***	0.185***	0.136***	0.247***	0.233***	0.007
	(4.503)	(3.061)	(3.735)	(4.578)	(3.884)	(0.142)
Age	0.641**	−0.522***	0.064	0.295***	−0.038	0.842***
	(2.465)	(−5.520)	(1.232)	(3.873)	(−0.438)	(11.732)
Year	Yes	Yes	Yes	Yes	Yes	Yes
Industry	Yes	Yes	Yes	Yes	Yes	Yes
Constant	−80.794***	−29.632***	−13.131***	−12.789***	−11.813***	−13.429***
	(−10.486)	(−5.333)	(−25.149)	(−15.862)	(−14.947)	(−11.792)
N	25,044	25,044	25,044	25,044	25,044	25,044
Adj. R-square	0.282	0.387	0.211	0.184	0.186	0.200

*** and ** indicate statistical significance at the 1 and 5% levels, respectively. The numbers in parentheses are t values; cluster-robust standard errors are used in model estimates to eliminate the heteroscedasticity effect.

Further, this paper subdivides CSR indicator into five dimensions: shareholder responsibility (*SH*), employee responsibility (*EM*), supply chain responsibility (*SCC*), environmental responsibility (*ENV*), and public responsibility (*SOC*). Columns (2)–(5) of Table 3 show the regression results of financial redundancy on various dimensions of CSR. The regression coefficients of financial redundancy (*FEX*) are significantly positively correlated with shareholder responsibility (*SH*) and public responsibility (*SOC*) at the level of 1%, but are significantly negatively correlated with employee responsibility (*EM*) and environmental responsibility (*ENV*) at the level of 1%, and are not significant with regard to supply chain responsibility (*SCC*). This finding shows that firms with financial redundancy still have the characteristics of “economic man,” and redundant resources aggravate the agency problem. Thus, even if enterprises have redundant financial resources, driven by self-interest motives they will be more willing to invest in social responsibility projects that can quickly bring profits to the firm. Employees and environmental responsibility will crowd out investment funds for other economic projects and reduce short-term performance. Therefore, firms with financial redundancy will not take the initiative to increase their responsibility investment in these aspects.

### Motivation Test

In this section, we aim to investigate the reason why firms with financially redundant resources toward better CSR practices compared to those without. According to the hypothetical derivation logic, the incentive for firms with financial redundancy to promote CSR initiatives may be attributable to their high level of social awareness and pursuit of reputation.

### The Social Awareness Motivation

Firms with financially redundant resources are usually large and subject to significant external attention. This means that their behavior is closely watched by the government, investors, media, and analysts. As external attention increases, corporate decisions become more complex and diverse because firms feel that their actions are being closely monitored and they must consider the interests of stakeholder groups in their decision-making process (Lindgreen et al., 2009; Jiang et al., 2022). Firms that receive large external attention are more inclined to display stronger social consciousness, which will encourage them to practice better CSR behavior.

To test whether firms that receive more attention will transfer public social awareness into corporate practices, we capture the social awareness of firms with financial redundancy using the interaction term of external attention and financial redundancy (*FEX × Attention*). External attention refers to the degree of attention paid to the firm by the external public, such as the government, investors, analysts, creditors, media, and consumers. In line with Cheng and Liu (2018), we use the natural logarithm of the internet search volume of Chinese listed companies as a proxy variable for external public attention to the firm. In column (1) of Table 4, the interaction term between financial redundancy and external attention is included, and its coefficient is positive and statistically significant, indicating that external attention drives enterprises to consider public interest and motivates them to invest available funds in socially responsible activities.

**TABLE 4:** Motivation test.

	(1)	(2)
	External attention	Pursuing reputation
Variables	CSR	CSR
FEX	1.635*	2.228***
	(1.884)	(3.709)
FEX × Attention	0.144**	
	(2.027)	
FEX × Reputation		12.633*
		(1.714)
Attention	0.231***	
	(12.618)	
Reputation		−2.537
		(−1.583)
State	2.097***	1.026***
	(9.127)	(4.625)
Size	1.906***	2.173***
	(20.364)	(23.412)
Roa	74.542***	64.951***
	(37.252)	(33.293)
Lev	−3.371***	−5.842***
	(−4.710)	(−8.390)
Growth	0.539***	0.138
	(2.658)	(0.691)
Top	4.157***	4.065***
	(6.606)	(6.682)
Salary	1.872***	3.237***
	(12.088)	(20.729)
Dual	−0.426**	−0.260
	(−2.254)	(−1.404)
Board	1.328***	0.806***
	(7.095)	(4.495)
Age	−2.491***	0.645**
	(−9.929)	(2.482)
Year	Yes	Yes
Industry	Yes	Yes
Constant	−46.015***	−80.297***
	(−20.458)	(−10.408)
N	25,044	25,044
Adj. R-square	0.234	0.282

***, **, and * indicate statistical significance at the 1, 5, and 10% levels, respectively. The numbers in parentheses are t values; cluster-robust standard errors are used in model estimates to eliminate the heteroscedasticity effect.

### The Reputation Motivation

Alternatively, firms with financial redundancy for CSR may stem from the desire of corporate executives to improve their reputation and social status through CSR engagement. Maslow’s hierarchy of needs theory points out that people pursue spiritual satisfaction once their material needs have been satisfied. Social responsibility has become an important tool to meet the needs of executives to enhance their social status and reputation (Barnea and Rubin, 2010; Masulis and Reza, 2015). Therefore, this paper attempts to explore whether corporate executives assign financially redundant resources to social responsibility activities to pursue reputation.

According to behavioral theory, the pay gap is an important part of social behavioral psychology, as it has a profound impact on whether executives pursue personal interests or actively achieve organizational goals. Drawing on Hou et al. (2021), this paper takes the percentage of executive compensation with total compensation as the standard to measure the degree of executives’ pursuit of reputation. The higher the value, the higher the monetary remuneration executives have received from the firm. Executives are more likely to pursue spiritual satisfaction when their profit needs are satisfied and are then more likely to use redundant resources to invest in social responsibility activities to pursue reputation. A smaller pay differential represents a greater incentive for executives to pursue economic benefits and deliberately avoid social responsibility. The results regarding this aspect are presented in column (2) of Table 4. The interaction term of *FEX × Reputation* is significant and positive, indicating that executives of firms with financially redundant resources are more inclined to use corporate funds to support their preference for social responsibility initiatives, and are willing to invest funds in such initiatives.

### Economic Consequences Test

The above findings confirm that financial redundancy can enhance CSR performance, but have yet to examine the ultimate impact of such promotion on firms. Therefore, it is necessary to further examine the economic consequences of financial redundancy on CSR to provide in-depth insights into motivations regarding CSR. The economic consequences of undertaking social responsibility include monetary and non-monetary benefits. Monetary benefits are measured according to corporate profitability, while non-monetary benefits are measured according to reputation. Below, we extend our study by exploring the incremental effects of CSR driven by financial redundancy from these two aspects.

First, we look into the interplay between financial redundancy and CSR performance on firm profitability. We empirically examine whether firms with significant financial redundancy can obtain profit-related benefits from enhanced CSR activities. To do so, we focus on Tobin’s q, which is a measure of a firm’s operating performance as a critical indicator, with the following specification:


(3) $$T\,Q_{i,t+1}=\delta_{1}+\delta_{2}\,F\,E\,X_{i,t}+\delta_{3}\,C\,S\,R_{i,t+1}+\delta_{4}\,F\,E\,X_{i,t}\times C\,S\,R_{i,t+1}+\sum_{j}\delta_{j}\,C\,o\,n\,t\,r\,o\,l_{i,t}+\varepsilon_{i,t}$$

Where *TQ* represents firm performance, using market value divided by total assets (Hou et al., 2021). The incremental effect of CSR through the presence of financial redundancy on firm profitability is captured by the interaction term between *FEX* and *CSR.* We control for several factors, including firm size, asset-liability ratio, the ratio of independent directors, the proportion of tangible asset, power balance with shareholder structure, firm age, and year and industry dummies. To the best of our knowledge, these factors can significantly affect corporate profitability. The results are shown in column (1) of Table 5. Among them, the coefficient of *FEX × CSR* is negative and significant at the level of 10%, indicating that increased CSR performance of firms with financial redundancy will not improve their profitability. As mentioned above, firms with financial redundancy will receive greater external attention. Firms participate in CSR to meet the expectations of, and pressure from, different stakeholders. In other words, firms with financial redundancy implement CSR behavior only in response to stakeholder pressure, and would not do so without such pressure. In this case, the stakeholder-driven motivation does not enhance corporate performance. This finding is consistent with empirical results found by Zhao et al. (2020). In addition, this result is in line with our subdivision indicators, in which we examine the impact of financial redundancy on different dimensions of social responsibility. Firms selectively undertake social responsibilities that can quickly improve short-term performance, and avoid social responsibilities that require long-term investment. Through the exchange mechanism, firms reduce some responsibilities in exchange for increasing others. However, this mode of strategically undertaking social responsibility will not increase corporate value.

**TABLE 5:** The incremental effect of CSR on profitability and corporate reputation.

	(1)	(2)
Variables	TQ	CR
FEX	3.762***	0.041***
	(2.919)	(3.496)
FEX × CSR	−0.056*	0.001**
	(−1.651)	(2.021)
CSR	0.000	0.001***
	(0.053)	(10.183)
Size	−0.475***	0.010***
	(−10.456)	(3.847)
Lev	2.022***	0.146***
	(4.386)	(15.140)
Tangible	0.255**	
	(2.145)	
Ebd	−0.001	
	(−1.541)	
Inde	0.016***	0.001***
	(4.180)	(5.392)
Age	0.518***	0.016***
	(4.668)	(4.294)
Roe		0.000
		(0.074)
MK		0.040**
		(2.484)
Year	Yes	Yes
Industry	Yes	Yes
Constant	7.756***	−0.158***
	(14.156)	(−8.189)
*N*	23,960	21,586
Adj. R-square	0.036	0.034

***, **, and * indicate statistical significance at the 1, 5, and 10% levels, respectively. The numbers in parentheses are t values; cluster-robust standard errors are used in model estimates to eliminate the heteroscedasticity effect.

Second, we examine the interaction between *FEX* and *CSR* on corporate reputation. The ranking method established by *Fortune* magazine in 1983, combines qualitative and quantitative reputation and is one of the most authoritative reputation ranking standards. However, the number of Chinese firms selected by *Fortune* magazine every year is small, and some non-listed firms are also included; thus, it provides insufficient data for this study. Fortunately, there are also authoritative institutions in China that evaluate the reputation of firms. *The economic observer*, an authoritative economic journal in China, provides a list of “China’s Most Respected Firms” every year, and the Chinese version of *Fortune* magazine also indicates “China’s Most Admired Firms”; these are two representative lists of corporate reputation in China. Therefore, by examining whether the sample firms are included on the above three lists, with 1 indicating inclusion and 0 otherwise, giving us a corporate reputation value ranging from 0 to 3. We then reestimate Equation 3 by replacing *TQ* with *CR*. Firm size, firm age, asset-liability ratio, return on equity, market power, the ratio of independent directors, year, and industry are selected as control variables. The result is displayed in column (2) of Table 5. The results clearly indicate that the coefficient on the interaction term, *FEX × CSR*, is positive and significant, which means that, on average, firms with more financially redundant resources perform better at propelling CSR practices, consequently gaining significant improvements in reputation. This conclusion also reflects the motivation of firms with financial redundancy to actively undertake social responsibility to gain reputation, improve social status, and pursue a sense of achievement.

Collectively, the results presented in this part imply that the enhanced CSR performance driven by financial redundancy improves corporate reputation, but has no enhancement effect on corporate performance. The economic consequences test further verifies the correctness of the hypothesis logic structure and the completeness of the motivation test. It also demonstrates that firms with financial redundancy strategically undertake social responsibility.

### Further Analysis

Previous studies have demonstrated that firms with available resources follow a specific investment sequence. To test the influence of other variables that may affect the relationship between financial redundancy and CSR, we examine how two key variables—managerial career concerns and market competition—affect the relationship between financial redundancy and CSR at the level of individual managers, as well as at the level of the external environment.

### Managerial Career Concerns

There is no doubt that socially responsible investment is made primarily based on senior management decisions (Zu and Song, 2009; Fabrizi et al., 2014). Studies of the relationship between financial redundancy and CSR have to take into account the decision-making role of managers. Upper echelons theory suggests that the subjective cognition and values of managers have a significant impact on corporate strategic decisions. According to career focus theory, managers consider the impact of their actions on future career prospects during the decision-making process. Career concerns shape managers’ perceptions and beliefs, and managers’ varying degrees of career concern directly affect their use of corporate resources and implementation of a CSR investment strategy. Drawing on Li et al. (2017), we divided our sample into high career concerns and low career concerns according to the average age of managers. Columns (1) and (2) of Table 6 show the relevant results. The results show that in the sample with high career concerns, the coefficient of *FEX* is positive and significant, while the coefficient of *FEX* fails the significance test in the sample with low career concerns. Thus, as corporate financial redundancy increases, the motivation of managers to make profitability their primary goal gradually weakens. Managers with high career concerns have a strong desire for achievement and reputation, which leads to young managers taking advantage of redundant resources to participate in social activities to gain a good social reputation. In contrast, managers with low career concerns tend to follow the firm’s existing strategy, make conservative decisions, and avoid engaging in the social responsibility practices.

**TABLE 6:** Further analysis.

	(1)	(2)	(3)	(4)
	High_career concerns	Low_career concerns	High_HHI	Low_HHI
Variables	CSR	CSR	CSR	CSR
FEX	10.620***	1.042	9.089***	0.657
	(14.006)	(1.464)	(13.384)	(0.793)
State	3.174***	2.729***	3.034***	2.632***
	(23.256)	(22.847)	(26.990)	(17.912)
Size	−8.957***	−14.681***	−10.172***	−14.479***
	(−9.676)	(−14.977)	(−11.968)	(−13.101)
Roa	1.330***	1.546***	1.286***	1.539***
	(5.015)	(4.871)	(4.972)	(4.813)
Lev	9.070***	4.508***	6.424***	4.919***
	(9.933)	(5.415)	(7.938)	(5.210)
Growth	3.060***	4.533***	3.714***	4.063***
	(13.530)	(20.726)	(18.165)	(16.739)
Top	−0.638**	−0.092	−0.749***	0.181
	(−2.530)	(−0.322)	(−3.023)	(0.635)
Salary	1.765***	0.368	0.721***	0.936***
	(5.925)	(1.602)	(3.132)	(3.247)
Dual	1.292***	0.638	1.076***	1.237***
	(3.658)	(1.613)	(2.993)	(3.279)
Board	10.620***	1.042	9.089***	0.657
	(14.006)	(1.464)	(13.384)	(0.793)
Age	3.174***	2.729***	3.034***	2.632***
	(23.256)	(22.847)	(26.990)	(17.912)
Year	Yes	Yes	Yes	Yes
Industry	Yes	Yes	Yes	Yes
Constant	−111.029***	−86.765***	−113.959***	−103.212***
	(−27.132)	(−23.397)	(−39.003)	(−29.594)
*N*	12,218	12,826	14,988	10,056
Adj. R-square	0.242	0.259	0.278	0.211

*** and ** indicate statistical significance at the 1 and 5% levels, respectively. The numbers in parentheses are t values; cluster-robust standard errors are used in model estimates to eliminate the heteroscedasticity effect.

### Market Competition

According to stakeholder theory, a firm’s stakeholders are those groups whose behaviors and interests are directly or indirectly affected by the firm’s behavior. Two categories of market participants are often discussed: primary stakeholders, which includes employees, customers, suppliers, distributors, competitors, shareholders, and state regulators; and secondary stakeholders, such as community activists, religious leaders, and non-governmental organizations. We argue that the two stakeholder groups will exert enormous pressure on firms through the market and require them to undertake corresponding social responsibilities. In turn, market competition will affect the relationship between financial redundancy and CSR. Our measure of market competition is the Herfindahl-Hirschman index (HHI), which is calculated by summing up the sales-based square market shares of all firms in the given industry. According to the industry median, the whole sample is divided into two groups: high market competition and low market competition. The results are shown in Table 6, columns (3) and (4), demonstrate that in the subsample regression of high market competition and low market competition, the coefficients of *FEX* are 9.089 and 0.657, respectively, with the latter failing the significance test. Findings show that the relationship between financial redundancy and CSR is influenced by degrees of pressure from a firm’s target market. The fiercely competitive environment prompts firms to engage in CSR practices because CSR can be used to differentiate themselves from competitors in the industry, and can also meet the needs of market stakeholders. The more competitive the market, the higher the level of CSR participation.

## Robustness Test

### Mitigating Potential Endogeneity Driven by Reverse Causality

Reverse causality may potentially bias our results because firms may obtain benefits from social responsibility activities, which in turn increases the level of financial redundancy. To rule out reverse causality as an alternative explanation for our results, following Dyck et al. (2019) and Li et al. (2021), we use the Granger causality test by estimating two symmetric sets of regressions; that is, we regress *CSR* on lagged *FEX* and lagged *CSR*, and *FEX* on lagged *CSR* and lagged *FE*X, with the same set of control variables, separately. The results are shown in Table 7, columns (1) and (2), and show that the coefficient of *FEX* is positive and significant, but that on *CSR* is not. Thus, we conclude that it is the financial redundancy that drives firms to engage in CSR practices, rather than firms reserving financial resources for social responsibility.

**TABLE 7:** Endogeneity test.

	(1)	(2)	(3)	(4)	(5)
				
	Granger causality test		PSM	Fixed-effects	Add control variables
				
Variables	CSR	FEX	FEX	CSR	CSR
FEX	3.059***	0.820***	3.094***	4.124***	2.691***
	(7.073)	(164.839)	(5.643)	(5.802)	(5.126)
CSR	0.536***	−0.000			
	(64.332)	(−0.005)			
State	0.206	0.014***	0.850***	−0.004	1.207***
	(1.091)	(7.959)	(3.769)	(−0.007)	(5.411)
Size	0.882***	−0.003***	2.232***	1.168***	2.178***
	(11.124)	(−3.953)	(24.124)	(5.986)	(24.042)
Roa	8.008***	0.050**	64.286***	21.667***	64.988***
	(4.255)	(2.369)	(31.602)	(10.657)	(33.169)
Lev	−2.725***	−0.036***	−5.980***	0.192	−5.627***
	(−4.568)	(−5.427)	(−8.309)	(0.183)	(−8.059)
Growth	0.563***	−0.006***	0.081	1.459***	0.125
	(3.180)	(−2.801)	(0.397)	(7.590)	(0.627)
Top	3.087***	0.017***	4.286***	4.585***	3.952***
	(5.953)	(3.344)	(6.809)	(3.784)	(6.465)
Salary	1.624***	0.003*	3.292***	1.958***	3.139***
	(12.309)	(1.902)	(20.360)	(7.665)	(19.887)
Dual	−0.180	−0.005***	−0.279	−0.584**	−0.326*
	(−1.130)	(−2.589)	(−1.446)	(−2.092)	(−1.754)
Board	0.304**	−0.002*	0.826***	0.249	0.790***
	(1.994)	(−1.875)	(4.490)	(0.999)	(4.313)
Age	0.449**	0.014***	2.067***	2.349**	0.700***
	(2.059)	(6.199)	(2.910)	(2.060)	(2.678)
RD					0.710
					(0.139)
ME					−5.297**
					(−2.186)
Inde					0.028*
					(1.726)
Market					1.528***
					(6.195)
Year	Yes	Yes	Yes	Yes	Yes
Industry	Yes	Yes	Yes	Yes	Yes
Firm	No	No	No	Yes	No
Constant	−47.328***	−0.060	−72.438***	−31.179***	−80.968***
	(−5.468)	(−1.482)	(−29.069)	(−5.938)	(−10.342)
*N*	25,044	25,044	23,512	25,044	25,030
Adj. R-square	0.476	0.788	0.285	0.052	0.283

***, **, and * indicate statistical significance at the 1, 5, and 10% levels, respectively. The numbers in parentheses are t values; cluster-robust standard errors are used in model estimates to eliminate the heteroscedasticity effect.

### Mitigating Potential Endogeneity Driven by Sample Selection Bias

Although the lag effect applied in this paper can alleviate certain endogeneity concerns, there may still be an endogeneity problem caused by sample selection bias. The propensity score matching (PSM) method is adopted to solve this problem. We construct a treatment group of financially redundant firms and a control group of non-financially redundant firms based on a common set of firm characteristics (*Size, Roa, Lev, Growth, Top, Salary, Age*), as well as the year, area, and industry dummies. This study uses the one-to-one nearest neighbor matching method. A total of 11,756 financially redundant samples are matched to 11,756 non-financially redundant samples with the most similar characteristics, and a total of 23,512 firm-year observations are obtained. Using the matched sample, this study reestimates Equation 1. The regression results are shown in column (3) of Table 7 and are qualitatively similar to those reported in column (1) of Table 3, which indicates that there is no substantial change in the conclusions of this paper.

### Mitigating Potential Endogeneity Driven by Omitted Variables

Although using an extensive list of control variables (in Equation 1) helps to reduce omitted variables bias in estimating the relationship between financial redundancy and CSR, the regression results still suffer from endogenous bias caused by unobserved omission variables. We used two methods of adding control variables and applying a fixed-effects model to mitigate potential endogeneity driven by omitted variables.

We use a fixed-effects model to control for relevant unobserved and observed time-invariant firm-specific factors that, if omitted from the model, often lead to considerable bias in the estimated results. The result is presented in column (4) of Table 7. Unsurprisingly, a positive and statistically significant relationship between financial redundancy and CSR still exists, which proves that our research conclusions have good robustness.

There is empirical evidence that research and development (R&D) expenditures are correlated with CSR choice (Padgett and Galan, 2010; Li et al., 2021). Similarly, the degree of marketization, the ratio of independent directors, and the management expense ratio are related to CSR investment (Flammer, 2015; Ardito et al., 2021; Shen et al., 2021). We additionally include regional marketization degree (*Market*), R&D expenditure (*RD*), the ratio of independent directors (*Inde*), and management expense ratio (*ME*) as control variables in Equation 1. The results are displayed in column (5) of Table 7. The coefficient of *FEX* remains positive and statistically significant at the conventional level, reaffirming the positive influence of financial redundancy on CSR despite the inclusion of some additional variables.

### Replacing Variables

We use additional methods to remeasure CSR to strengthen the conclusions. Specifically, we apply three methods that are commonly used in academia to remeasure CSR. First, the CSR rating given by Hexun is used instead of the score. The higher the rating, the better the CSR performance. The ratings are A to E, so the *CSR* variable is assigned values 5 to 1 accordingly. Second, the social contribution per share is used to measure the CSR performance of listed firms. Third, the difference between the next year’s CSR score and the current year’s CSR score is used to measure CSR. Equation 1 is tested by remeasuring CSR through the above three methods. The results in columns (1)–(3) of Table 8 imply that the coefficients of *FEX* are always positive and significant even if the measurement method of CSR is changed.

**TABLE 8:** Replacing variables.

	(1)	(2)	(3)	(4)	(5)
Variables	CSR	CSR	CSR	CSR	CSR
FEX	0.038**	1.294***	3.410***	1.079***	2.868***
	(1.974)	(10.945)	(7.103)	(4.910)	(5.496)
State	0.056***	0.057	−0.515**	1.019***	1.032***
	(6.587)	(1.350)	(−2.411)	(4.587)	(4.650)
Size	0.065***	0.297***	−0.266***	2.191***	2.208***
	(20.181)	(14.875)	(−2.970)	(24.328)	(24.451)
Roa	0.154***	7.251***	−41.217***	65.264***	64.722***
	(2.858)	(19.022)	(−22.566)	(33.651)	(33.231)
Lev	−0.218***	1.517***	−0.012	−6.429***	−5.705***
	(−8.580)	(12.458)	(−0.018)	(−9.798)	(−8.216)
Growth	−0.000***	0.039	0.940***	0.130	0.127
	(−4.262)	(1.326)	(4.786)	(0.650)	(0.635)
Top	0.055**	0.340***	2.238***	4.135***	4.062***
	(2.408)	(3.197)	(3.830)	(6.807)	(6.676)
Salary	0.086***	0.154***	0.250*	3.239***	3.203***
	(14.752)	(3.080)	(1.700)	(20.855)	(20.587)
Dual	−0.006	0.097***	−0.121	−0.237	−0.250
	(−0.831)	(2.622)	(−0.674)	(−1.282)	(−1.352)
Board	0.024***	0.043*	−0.133	0.801***	0.809***
	(3.491)	(1.841)	(−0.771)	(4.467)	(4.513)
Age	0.014	−0.032	0.284	0.608**	0.646**
	(1.401)	(−0.774)	(1.167)	(2.342)	(2.485)
Year	Yes	Yes	Yes	Yes	Yes
Industry	Yes	Yes	Yes	Yes	Yes
Constant	−2.091***	−10.006***	−18.325*	−80.126***	−79.052***
	(−20.847)	(−16.439)	(−1.904)	(−10.432)	(−10.392)
N	25,044	16,349	25,044	25,044	25,044
Adj. R-square	0.181	0.138	0.101	0.282	0.282

***, **, and * indicate statistical significance at the 1, 5, and 10% levels, respectively. The numbers in parentheses are t values; cluster-robust standard errors are used in model estimates to eliminate the heteroscedasticity effect.

We also replace the measure of financial redundancy. We use dummy variables to remeasure financial redundancy. If the sum of monetary funds and transactional financial assets of the firm is greater than the total amount of interest-bearing liabilities of the firm, *FEX* is coded as 1, and 0 otherwise. Equation 1 is retested and the results are presented in column (4) of Table 8. In addition, considering that industry characteristics may affect corporate financial redundancy, referring to Harford et al. (2008), the industry-adjusted financial redundancy index is used as a proxy for financial redundancy to conduct robustness tests, and the results are shown in column (5). In both cases, the evidence reconfirms our main findings.

In sum, our results are robust after a series of replacement variable measures and further confirm that financial redundancy plays an important role in promoting CSR activities.

## Conclusion

This study takes Chinese A-share listed firms from 2010 to 2020 as the research object. Based on stakeholder theory, resource slack theory, agency cost theory, and behavioral theory, the influence of financial redundancy on CSR is discussed from the perspective of social responsibility as a whole, and social responsibility from various dimensions. The main results are as follows: (1) firms with financial redundancy have significantly higher CSR concerns vs. those without such resources, that is to say, financial redundancy significantly improves their overall CSR performance. This result remains following a series of robustness tests. (2) Through the decomposition of social responsibility indicators, it can be found that firms with financial redundancy give priority to strengthening their social responsibility performance in relation to shareholders and the public, while ignoring their responsibility to employees and the environment. (3) By examining the motivations and economic consequences of CSR, we show that firms with financial redundancy take on more social responsibility to meet social needs and obtain reputation, which further reveals that firms undertaking social responsibility is a strategic behavior. (4) Further analysis shows that the positive impact of financial redundancy on CSR is more significant in firms with high managerial career concerns and high market competition than firms with low managerial career concerns and low market competition.

Our findings have important implications for enterprises and governments. For enterprises, on the one hand, firms leaders should establish a scientific perception of social responsibility. Although the fulfillment of social responsibilities entails financial cost, the motivation for enterprises to fulfill their social responsibilities should not only be self-interested as a profit-making tool, but should also embed social responsibility missions into corporate strategies and business operations. Choosing to assume social responsibilities for short-term effects and avoiding social responsibilities that can bring long-term effects neither brings lasting economic benefits nor become competitive advantage to the firm. The firm needs to incorporate multidimensional social responsibility into corporate strategic considerations. On the other hand, companies need to establish a good corporate governance mechanism. Companies can reduce agency problems by separating the position of chairman and CEO, increasing the size of directors’ ownership structure, and providing appropriate incentives to senior management. Stimulating the decision-making of enterprise managers and shareholders reflect the needs of stakeholders, thereby preventing and curbing all kinds of misconduct that may damage the interests of enterprises. The government needs to improve the external institutional environment. Catering to social needs and reputation mechanisms are the motivations for financially redundant firms to fulfill their social responsibilities. If the government further promulgates policies to strengthen the necessity for enterprises to undertake social responsibility and strengthens the supervision role of media on corporate social responsibility behaviors, which can help enterprises to establish standardized social responsibility content and boundary.

## Data Availability Statement

The raw data supporting the conclusions of this article will be made available by the authors, without undue reservation.

## Author Contributions

LH chose the research ideas, formed the study framework, and wrote the manuscript. SG collected and analyzed the data. TZ reviewed and edited the manuscript. All authors read and approved the final manuscript.

## Conflict of Interest

The authors declare that the research was conducted in the absence of any commercial or financial relationships that could be construed as a potential conflict of interest.

## Publisher’s Note

All claims expressed in this article are solely those of the authors and do not necessarily represent those of their affiliated organizations, or those of the publisher, the editors and the reviewers. Any product that may be evaluated in this article, or claim that may be made by its manufacturer, is not guaranteed or endorsed by the publisher.

## Footnotes

1
http://stockdata.stock.hexun.com
